# A systematic review and meta-analysis of the SIRT1 response to exercise

**DOI:** 10.1038/s41598-023-38843-x

**Published:** 2023-09-07

**Authors:** Ciara Gallardo Juan, Kyle B. Matchett, Gareth W. Davison

**Affiliations:** 1 Sport and Exercise Sciences Research Institute, Ulster University, Belfast, BT15 1AP UK; 2 Personalised Medicine Centre, School of Medicine, Ulster University, Derry/Londonderry, BT47 6SB UK

**Keywords:** Ageing, DNA, Biochemistry, Cell biology

## Abstract

Sirtuin 1 (SIRT1) is a key physiological regulator of metabolism and a target of therapeutic interventions for cardiometabolic and ageing-related disorders. Determining the factors and possible mechanisms of acute and adaptive SIRT1 response to exercise is essential for optimising exercise interventions aligned to the prevention and onset of disease. Exercise-induced SIRT1 upregulation has been reported in animals, but, to date, data in humans have been inconsistent. This exploratory systematic review and meta-analysis aims to assess various exercise interventions measuring SIRT1 in healthy participants. A total of 34 studies were included in the meta-analysis (13 single bout exercise, 21 training interventions). Studies were grouped according to tissue sample type (blood, muscle), biomarkers (gene expression, protein content, enzyme level, enzyme activity), and exercise protocols. A single bout of high-intensity or fasted exercise per se increases skeletal muscle *SIRT1* gene expression as measured by qPCR or RT-PCR, while repeated resistance training alone increases blood SIRT1 levels measured by ELISA. A limited number of studies also show a propensity for an increase in muscle SIRT1 activity as measured by fluorometric or sirtuin activity assay. In conclusion, exercise acutely upregulates muscle *SIRT1* gene expression and chronically increases SIRT1 blood enzyme levels.

## Introduction

Sirtuins, dubbed as cellular ‘*watchmen*’ and ‘*stress sensors*’, control cell function by determining cell fate, maintaining energy supply, and preventing DNA damage to maintain genomic integrity. The mammalian sirtuin family of enzymes, composed of sirtuins 1–7 (SIRT1-SIRT7), have received considerable interest due to their role in regulating responses aligned to physiological stress and thus, health and longevity. Sirtuins function as histone deacetylase (HDAC), removing acetyl groups from target proteins and effectively activating or inhibiting these proteins depending on the specific cellular context^1^. For sirtuins to work efficiently, they use nicotinamide adenine dinucleotide (NAD^+^), through a reduction and oxidation mechanism which fuels the synthesis of ATP^2^. NAD^+^ and its redox couples, NADH and NADP(H), not only control metabolism and sirtuins, but also regulate several redox-sensitive pathways^3^. Sirtuins, in turn, regulate these redox pathways directly through deacetylation and indirectly by maintaining the NAD^+^ pool.

SIRT1 is known to deacetylate a range of salient transcription factors and proteins, including: AMP-activated protein kinase (AMPK), a central regulator of energy metabolism that maintains cell ATP concentration; peroxisome proliferator-activated receptor alpha (PPAR-α) involved in lipid metabolism; peroxisome proliferator-activated receptor gamma coactivator 1-alpha (PGC-1α) involved in mitochondrial metabolism; nuclear factor erythroid 2–related factor 2 (Nrf2), an antioxidant transcription factor; nuclear factor kappa-light-chain-enhancer of activated B cells (NF-κB), the regulator of innate immunity; and Ku70, p53, forkhead box transcription factors (FOXO) that are involved in DNA repair and cell survival in ageing and cancer^1,4^. In cancer for example, the balance between inhibitory SIRT1 deacetylation of p53 and SIRT1 recruitment of p53 acetylation, can determine whether damaged cells survive or undergo apoptosis^5^. SIRT1 also enhances cell survival in the heart-brain axis by upregulating brain-derived neurotrophic factor (BDNF) that is crucial for neuronal growth, synaptic plasticity, and vascular endothelial growth factor (VEGF) signalling^6^.

In addition to transcription factors, SIRT1 deacetylates histones—structures around which DNA wraps—facilitating chromatin compaction and silencing of genes involved in disease. For example, SIRT1 deacetylates histones at H4K16, decreasing its binding with the pro-inflammatory cytokine TNF-α promoter and alleviating inflammation^7^. Histone deacetylation is also involved in SIRT1’s regulation of circadian genes that control the production of hormones and enzymes, including nicotinamide phosphoribosyltransferase (NAMPT), the rate-limiting enzyme in NAD synthesis^8,9^. Hence, loss of SIRT1 results in reduced NAD, impaired circadian rhythm, and increased expression of ageing-related genes^10^.

Ageing is characterised by decreased SIRT1, NAMPT, NAD^+^, and changes in circadian rhythm^9^. This regulatory network forms the basis of developing SIRT1 activators and NAD^+^ precursors for ageing-related disorders. In old mice, supplementation with NAD^+^ precursors can increase SIRT1 activity, stem cell regeneration, mitochondrial and physical function, and lifespan^11^. Similarly, SIRT1 overexpression can delay ageing, restore physical fitness, and extend lifespan in old mice^12^. Exercise-induced SIRT1 upregulation has been shown to decrease inflammation, apoptosis, and metabolic dysfunction in mice^13–15^. Similarly, in humans, aerobic exercise-induced SIRT1 increases antioxidant capacity (catalase and superoxide dismutase mainly) while decreasing cell senescence in heart failure^16,17^, and improves overall metabolic profile in type 2 diabetes^18^.

ATP demand during high-intensity exercise increases the AMP:ATP ratio that is sensed by AMPK, which responds by stimulating cell glucose uptake and fatty acid oxidation to generate more ATP and NAD^+^, the latter serving as a fuel for SIRT1^19^. Exercise also generates reactive oxygen species (ROS) such as H_2_O_2_, a known signalling molecule that activates AMPK as a secondary consequence of mitochondrial ATP production^20^. However, excessive ROS can also repress SIRT1 activity by post-translational oxidative modification or by modifying intracellular NAD^+^ levels^21^. Indeed, high-intensity exercise increases DNA damage, which can recruit other NAD^+^-consuming repair enzymes such as poly ADP ribose polymerases (PARPs) that compete with and inhibit SIRT1^21,22^. As the SIRT1 response to exercise is complex, it is essential to ascertain how exercise intensity, type, or duration can affect SIRT1 levels, both acutely (single bout) and after repeated training. Therefore, the aim of this exploratory review is to summarise and systematically assess published exercise interventions quantifying SIRT1 (protein content, gene expression, enzyme levels, and enzyme activity) in apparently healthy participants. Specifically, our primary objective is to determine if exercise can increase SIRT1, with a further objective to ascertain the type of exercise that may cause any modification to SIRT1.

## Results

### Literature search

**Figure 1:**
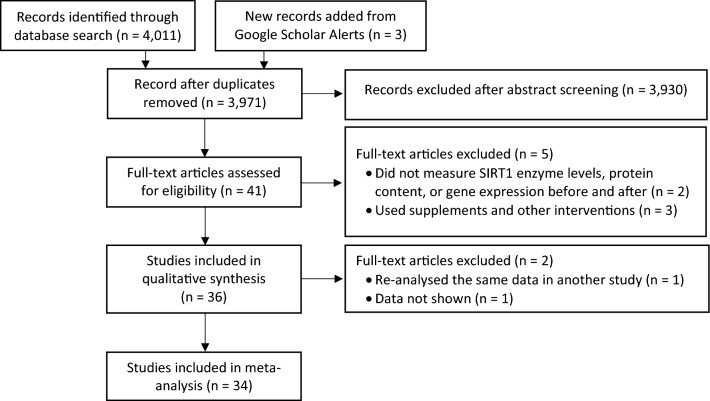
PRISMA flow diagram showing the database search and selection process.

A database search retrieved a total of 3,971 non-duplicate articles, from which 34 were included in the meta-analysis (13 acute response and 21 training interventions). The search and selection process is summarised in Fig. 1.

### Study characteristics

#### Participants

Participant age ranged from 20–66 years old. Six studies involved seniors, six studies tested overweight/obese participants, and the remaining studies involved young to middle-aged, normal weight participants. Only 10 studies involved women.

#### Quality assessment of individual studies

Studies scored 6 or higher, which is within the threshold for separating high-quality from low-quality studies based on a validity study of the original 11-item Cochrane Back Group Risk of Bias Tool^23^.

#### Biomarkers/analytical techniques

Studies were classified according to tissue and biomarker: *SIRT1* gene expression in skeletal muscle (measured via qPCR or RT-PCR), SIRT1 protein content in skeletal muscle (measured via Western blot), SIRT1 enzyme levels in blood (measured via ELISA), and SIRT1 enzyme activity in skeletal muscle (measured via fluorometric or sirtuin activity assay).

#### Exercise classification

Studies were also grouped based on exercise type, intensity, duration, and feeding status of participants (fasted or fed). “Fasted” was used to accurately describe the exercise protocols that implemented an overnight fast, however, we cannot determine the effects of fasting on exercise per se since the said studies did not include a non-fasted control group. Intermediate and advanced yoga and Pilates interventions were included and classified as resistance training, while meditative yoga interventions consisting of breathing exercises alone were excluded. Table 1 summarises all exercise studies used in the meta-analysis and their abbreviations, while the exact protocols used are summarised in Tables 2 and 3.

**Table 1:** Exercise classification table based on type, intensity, duration, tissue sample, biomarker, and feeding status of participants.

	Type, intensity	Feeding status	Tissue	Biomarker
Acute response studies				
Aird et al., 2021^25^	SIT	Fasted	Muscle	Gene expression
Cho et al., 2022^26^	MIA, HIA	Fed	Blood	Enzyme levels
Dumke et al., 2009^27^	EnA	Fed	Muscle	Gene expression
Edgett et al., 2013^28^	HIIT	Fed	Muscle	Gene expression
Ghasemi et al., 2020^29^	SIT	Fed	Blood	Enzyme levels
Granata et al., 2020^30^	HIIT	Fed	Muscle	Gene expression
Guerra et al., 2010^31^	SIT	Fasted	Muscle	Protein content
Margolis et al., 2017^32^	EnA	Fasted	Muscle	Gene expression
Morales-Alamo et al., 2013^33^	SIT	Fasted	Muscle	Protein content
Mendham et al., 2016^34^	MIA	Fasted	Muscle	Protein content
Potthast et al., 2020^35^	HIA	Fed	Blood	Enzyme activity
Radak et al., 2011^36^	HIA	Fasted	Muscle	Gene expression
Skelly et al., 2017^37^	SIT	Fed	Muscle	Gene expression
Intervention studies				
Afzalpour et al., 2017^38^	HIIT		Blood	Enzyme levels
Alfieri et al., 2015^39^	MIA		Muscle	Gene expression
Amirsasan et al., 2019^40^	RT		Blood	Enzyme levels
Boyd et al., 2013^41^	HIIT		Muscle	Protein content
Dimauro et al., 2016^42^	RT		Blood	Enzyme levels
Ghasemi et al., 2020^29^	HIIT		Blood	Enzyme levels
Gliemann et al., 2013^43^	HIIT		Muscle	Protein content
Granata et al., 2020^30^	HIIT		Muscle	Gene expression
Gray et al., 2018^44^	SIT		Blood	Gene expression
Gurd et al., 2010^45^	HIIT		Muscle	Enzyme activity protein content
Gurd et al., 2011^46^	HIIT		muscle	Enzyme activity protein content
Hooshmand-Moghadam et al., 2020^47^	RT		Blood	Enzyme levels
Kababi et al., 2022^48^	RT		Blood	Enzyme levels
Lamb et al., 2020^49^	RT		Muscle	Enzyme activity protein content
Little et al., 2010^50^	HIIT		Muscle	Protein content
Ma et al., 2013^51^	HIIT		Muscle	Protein content
Scribbans et al., 2014^52^	HIIT		Muscle	Gene expression
Soltani et al., 2018^53^	HIA		Blood	Enzyme levels
Skleryk et al., 2013^54^	SIT		Muscle	Protein content
Tolahunase et al., 2017^55^	RT		Blood	Enzyme levels
Wasserfurth et al., 2021^56^	RT		Blood	Gene expression enzyme activity

*EnA* endurance aerobic*, HIA* high-intensity aerobic*, HIIT* high-intensity interval training, *MIA* moderate-intensity aerobic, *RT* resistance training, *SIT* sprint interval training.

**Table 2:** Effects of a single bout of exercise on SIRT1.

Study	Participants	Exercise protocol	Sample	Biomarker	Technique	Post-exercise SIRT1 (vs rest)
Aird et al., 2021^25^	Recreationally active males (N = 9, 26.1 ± 5.1 years)	SIT (4 × 30 s all-out cycle sprints against resistance of 75% BM, at > 80% VO_2max_) on overnight fast	Muscle tissue	*SIRT1* mRNA gene expression	Multiplex PCR	↑ at 3 h
Cho et al., 2022^26^	Young men (MIA N = 10, HIA N = 10, 20.70 ± 1.34 years)	Treadmill MIA (65% VO_2max_) and HIA (85% VO_2max_)	Blood	SIRT1 enzyme levels	ELISA	↑ in both conditions
Dumke et al., 2009^27^	Trained male cyclists (N = 40, 29.1 ± 2.4 years)	3 h of cycling at ≈57% W_max_	Muscle tissue	*SIRT1* mRNA gene expression	qPCR	↑ at 0 h
Edgett et al., 2013^28^	Recreationally active males (N = 8 in each intensity, 21.9 ± 2.2 years)	HIIT (cycling at 11 × 60 s at 73% WR_peak_, 8 × 60 s at 100% WR_peak_, or 6 × 60 s at 133% WR_peak_)	Muscle tissue	*SIRT1* mRNA gene expression	RT-PCR	↑ at 3 h in all conditions
Ghasemi et al., 2020^29^	Overweight women (trained N = 10, untrained N = 10, 23.58 ± 2.23 years)	Wingate test (4 × 30 s all-out cycling at .075 kg/kg BM)	Blood	Serum SIRT1	ELISA	↑ in trained No significant effect in untrained
Granata et al., 2020^30^	Males (trained N = 8, untrained N = 8, 20 ± 2 years)	HIIT (5 × 4 min cycling at ≈107.4% of Ẇ_LT_)	Muscle tissue	*SIRT1* mRNA gene expression	qPCR	↓ at 0 h in both conditions
Guerra et al., 2010^31^	Male P.E. students (N = 8, 23.4 ± .6 years)	30 s Wingate test at 100 rpm, ≈120% VO_2max_ on overnight fast	muscle tissue	SIRT1 protein content	Western blot	↑ at 2 h
Margolis et al., 2017^32^	Physically fit men and women (cycling group N = 7, treadmill group N = 5, 22 ± 1 years)	1.5 h of of cycling or loaded treadmill walk at ≈58% VO_2peak_ on overnight fast	Muscle tissue	*SIRT1* mRNA gene expression	RT-PCR	↑ at 0 h and 3 h in both groups
Morales-Alamo et al., 2012	Male P.E. students (N = 10, 25 ± 4 years)	30 s Wingate test at 100 rpm, ≈120% VO_2max_ on overnight fast	Muscle tissue	SIRT1 protein content	Western blot	No significant effect
Morales-Alamo et al., 2013^33^	Male P.E. students (N = 9, 25 ± 5 years)	30 s Wingate test at 100 rpm, ≈120% VO_2max_ on overnight fast	Muscle tissue	SIRT1 protein content	Western blot	No significant effect
Mendham et al., 2016^34^	Sedentary, obese, middle-aged men (N = 9 rugby, N = 9 cycling, 48.8 ± 1.7 years)	40 min of touch rugby or cycling at RPE = 13–14 on overnight fast	Muscle tissue	SIRT1 protein content	Western blot	No significant effect in either condition
Potthast et al., 2020^35^	Recreational runners (N = 25, 27.2 ± 4.1 years)	GXT (16.7 W per minute) until voluntary exhaustion on bicycle	Blood	SIRT1 activity	Sirtuin activity assay	↑ at 0 h
Radak et al., 2011^36^	Young sedentary (N = 6, 26.0 ± 4.5 years), young physically active (N = 6, 30.2 ± 7.9 years), old sedentary (N = 6, 63.4 ± 4.7 years), and old physically active (N = 6, 62.4 ± 2.9)	45 min of treadmill run at 70–75% VO_2max_ then increased to 90%VO_2max_ and terminated at exhaustion, on overnight fast	Muscle tissue	*SIRT1* mRNA gene expression	RT-PCR	↑ at 0 h in young sedentary ↑ at 0 h in young active ↑ at 0 h in old sedentary No significant effect in old active
Skelly et al., 2017^37^	Sedentary participants (men N = 8, women N = 8, 22 ± 3 years)	SIT (3 × 20 s all-out cycling at ≈ 500W)	Muscle tissue	*SIRT1* mRNA gene expression	RT-PCR	↑ at 3 h in men ↑ at 3 h in women

*BM* body mass, *GXT* graded exercise test, *HIIT* high-intensity interval training, *HR*_*max*_ maximum heart rate*, RPE* rating of perceived exertion using Borg scale*, rpm* revolutions per minute, *SIT* sprint interval training, *W* watts, *W*_*max*_ maximal power, *W*_*peak*_ peak power, *W*_*LT*_ power at lactate threshold, *WR*_*peak*_ peak work rate, ↑ increase, ↓ decrease.

**Table 3:** Effects of repeated exercise training on SIRT1.

Study	Participants	Training Intervention	Sample	Biomarker	Technique	Resting SIRT1 Post-Training (vs Pre-Training)
Afzalpour et al., 2017^38^	Overweight women (N = 10, 20–25 years)	HIIT (85–95% HR_max_) 3 times/week for 10 weeks	Blood	SIRT1 enzyme levels	ELISA	↑
Alfieri et al., 2015^39^	Untrained males (N = 5, 20–43 years)	1 h of football training for 64 weeks (2.4 times/week for the first 12 weeks, and 1.3 times/week for the following 52 weeks)	Muscle Tissue	*SIRT1* gene expression	RT-PCR	↑
Amirsasan et al., 2019^40^	Sedentary overweight middle-aged women (N = 12)	Pilates with weights and bands, 3 times/week for 12 weeks	Blood	SIRT1 enzyme levels	ELISA	↑
Boyd et al., 2013^41^	Sedentary overweight/obese males (N = 10 moderate-intensity, N = 9 high-intensity, 22.7 ± 3.9 years)	Progressive interval cycling (8–10 × 60 s at either 70% or 100% WR_peak_) 3 times/week for 3 weeks	Muscle Tissue	Whole muscle SIRT1 protein content	Western blot	↑ in both conditions
Dimauro et al., 2016^42^	Senior men and women (N = 10)	Explosive resistance training (70% 1RM) 2 times/week for 12 weeks	Blood	SIRT1 protein levels	Western blot	No significant effect
Ghasemi et al., 2020^29^	Sedentary overweight women (N = 10, 23.58 ± 2.23 years)	HIIT (shuttle run at 90% HR_max_) 3 times/week for 10 weeks	Blood	SIRT1 enzyme levels	ELISA	↑
Gliemann et al., 2013^43^	Physically inactive senior men (N = 13, 65 ± 1 years)	HIIT on bicycle twice/week, CrossFit once a week, and 5 km walk once a week, for 8 weeks	Muscle Tissue	SIRT1 protein content	Western blot	No significant effect
Granata et al., 2020^30^	Moderately trained males (N = 8, 20 ± 2 years)	Progressive HIIT (5–12 × 4 min or 8–22 × 2 min cycling intervals at up to ≈98.8% of Ẇ_LT_) twice a day for 20 days	Muscle Tissue	*SIRT1* mRNA gene expression	qPCR	No significant effect
Gray et al., 2018^44^	Recreationally active men and women (N = 20, 22.8 ± 2.8 years)	SIT (4–6 × 30 s maximal cycling sprints at a resistance of 7.5% BM) 3 times/week for 4 weeks	Blood	*SIRT1* mRNA gene expression	qPCR, hSIRTNADPlex assay	No significant effect
Gurd et al., 2010^45^	Recreationally active men and women (N = 9, 23.4 ± 1.1 years)	HIIT (∼1 h of 10 × 4 min cycling intervals at 90% VO_2peak_) 3 times/week for 6 weeks	Muscle Tissue	Total SIRT1 activity, intrinsic activity per SIRT1 protein in muscle, SIRT1 protein content	Western blot, fluorometric assay	↑ total activity ↑ intrinsic activity ↓ content
Gurd et al., 2011^46^	Recreationally active men and women (N = 7, 23.4 ± 1.1 years)	HIIT (10 × 4 min cycling intervals at 90% VO_2peak_) 3 times/week for 2 weeks	Muscle Tissue	Whole muscle and nuclear SIRT1 protein content, nuclear SIRT1 activity	Western blot, fluorometric assay, qPCR	No significant change in whole muscle and nuclear SIRT1 content ↑ nuclear activity
Hooshmand-Moghadam et al., 2020^47^	Untrained senior men (N = 15, 66.33 ± 3.35 years)	Progressive full-body resistance training (4 × 15 tempo repetitions per muscle group at 60% 1RM) 3 times/week for 12 weeks	Blood	SIRT1 enzyme levels	ELISA	↑
Kababi et al., 2022^48^	Male athletes (N = 10)	Progressive lower-body resistance training (30–70% 10RM) for 12 weeks	Blood	SIRT1 enzyme levels	ELISA	No significant effect
Lamb et al., 2020^49^	Untrained overweight middle-aged (N = 16)	Full-body resistance training 2 times/week for 10 weeks	Muscle Tissue	SIRT1 protein content, activity	Western blot, SIRT1 activity assay	No significant effect in content ↑ activity
Little et al., 2010^50^	Recreationally active men (N = 7, 21 ± 1 years)	Progressive HIIT (8–12 × 60 s cycling intervals at W_peak_ (355 ± 10 W) 3 times/week for 2 weeks	Muscle Tissue	SIRT1 protein content	Western blot	↑
Ma et al., 2013^51^	Recreationally active men (N = 8, 20.6 ± 1.6 years)	Tabata protocol (8 × 20 s cycling intervals at 170% WR_peak_) 4 times/week for 4 weeks	Muscle Tissue	Whole muscle SIRT1 protein content	Western blot	No significant effect
Scribbans et al., 2014^52^	Recreationally active men (N = 8, 21 ± 1 years)	Tabata protocol (8 × 20 s cycling intervals at 170% WR_peak_) 3 times/week for 4 weeks	Muscle Tissue	*SIRT1* gene expression	qPCR	No significant effect
Skleryk et al., 2013^54^	Sedentary obese men (N = 8 sprint, N = 8 traditional exercise, 37.8 ± 5.8 years)	SIT (6 sessions of 8–12 × 10 s sprints) or traditional exercise (10 sessions of 30 min cycling) at 65% VO_2peak_ in a span of 2 weeks	Muscle Tissue	SIRT1 protein expression	Western blot	No significant effect in either intervention
Soltani et al., 2018^53^	Obese men (N = 11)	Water training (60–80% HR_max_) 3 times/week for 8 weeks	Blood	SIRT1 enzyme levels	ELISA	↑
Tolahunase et al., 2017^55^	Young, middle-aged, and seniors (N = 94)	Progressive yoga 5 times/week for 12 weeks	Blood	SIRT1 enzyme levels	ELISA	↑
Wasserfurth et al., 2021^56^	Untrained seniors (N = 14, 60 ± 6 years)	Progressive strength endurance circuit (full-body strength exercises with machines and 2 × 4 min bouts on bicycle and cross-trainer) at RPE = 15, twice/week for 12 weeks	Blood	*SIRT1* gene expression, SIRT1 activity	RT-PCR, fluorometric assay	No significant effect in expression ↑ activity

*BM* body mass, *HIIT* high-intensity interval training, *HR*_*max*_ maximum heart rate, *RPE* rating of perceived exertion using Borg scale, *RM* repetition maximum, *SIT* sprint interval training, *VO*_*2peak*_ peak oxygen consumption*, WR* work rate, *W* watts, *W*_*peak*_ peak power, *WR*_*peak*_ peak work rate, ↑ increase, ↓ decrease.

### Acute SIRT1 response to exercise

**Figure 2:**
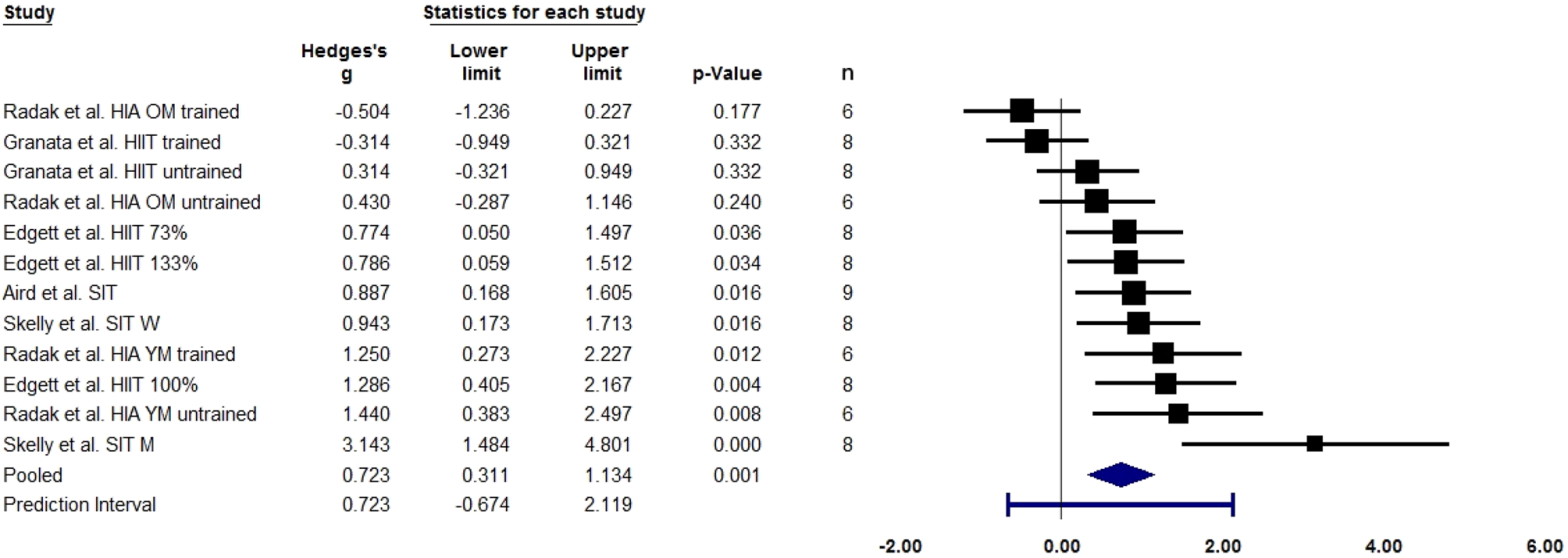
Forest plot quantifying skeletal muscle *SIRT1* gene expression (measured via qPCR or RT-PCR) following a single bout of high-intensity exercise. Adjusted standardised mean difference (Hedges’ g), relative weight of each acute study response, confidence interval (diamond), and prediction interval (blue line) are also shown. *HIA* high-intensity aerobic, *HIIT* high-intensity interval training, *OM* old men, *M* men, *SIT* sprint interval training, *W* women, *YM* young men. Percentages denote proportion of exercise intensity. n = sample size.

**Figure 3:**
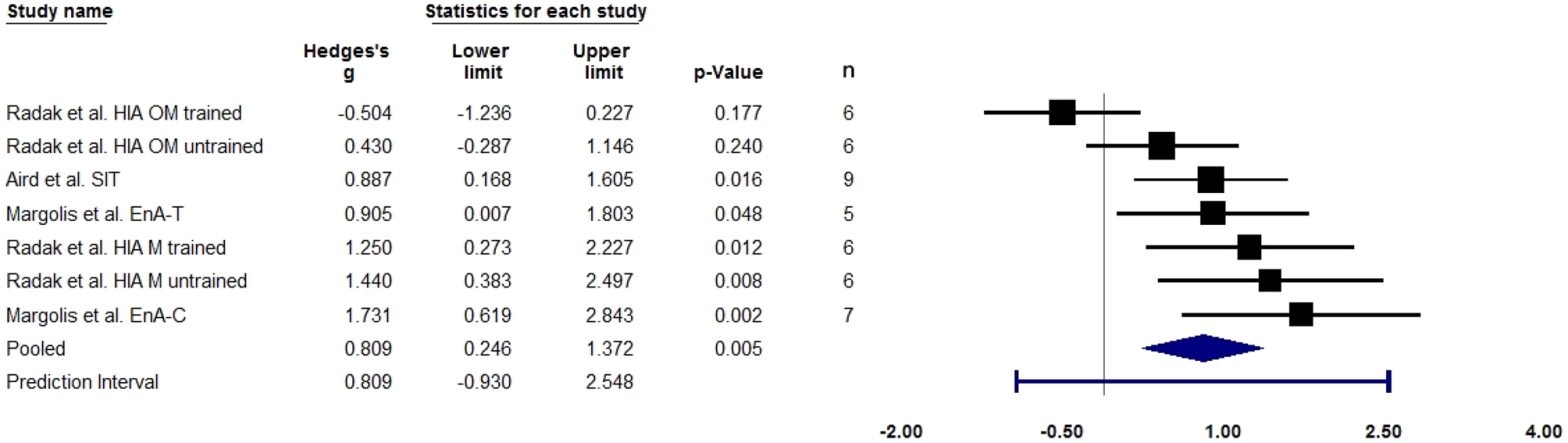
Forest plot quantifying skeletal muscle *SIRT1* gene expression (measured through qPCR or RT-PCR) following a single bout of fasted exercise (overnight fast). Adjusted standardised mean difference (Hedges’ g), relative weight of each acute study response, confidence interval (diamond), and prediction interval (blue line) are also shown. *EnA-C* endurance aerobic cycling, *EnA-T* endurance aerobic treadmill, *HIA* high-intensity aerobic, *OM* old men, *M* men, *SIT* sprint interval training. n = sample size.

Skeletal muscle *SIRT1* gene expression (measured via qPCR or RT-PCR) increased after a single bout of high-intensity exercise or following fasted exercise. Study characteristics are summarized in Figs. 2 and 3.

#### High-intensity exercise

The analysis is based on 12 studies and utilised a random-effects model. The mean effect size adjusted with Hedges’ g is 0.723 with a 95% confidence interval of 0.311–1.134. To test the null hypothesis that the mean effect size is zero, we used the Z-value which is 3.444 with p = 0.001, and using a criterion alpha of 0.050, we reject the null hypothesis and conclude that in the universe of populations comparable to those in the analysis, the mean effect size is not precisely zero. To test the null hypothesis that all studies in the analysis share a common effect size, we used the Q-value which is 35.016 with 11 degrees of freedom and p < 0.001, and using a criterion alpha of 0.100, we reject the null hypothesis that the true effect size is the same in all these studies. The I-squared statistic is 69%, which suggests that some 69% of the variance in observed effects reflects variance in true effects rather than sampling error. Assuming that the true effects are normally distributed, we can estimate that the prediction interval is − 0.674 to 2.119. The true effect size in 95% of all comparable populations falls between this interval.

#### Fasted exercise

The analysis is based on seven studies and utilised a random-effects model. The mean effect size adjusted with Hedges’ g is 0.809 with a 95% confidence interval of 0.246–1.372. The Z-value is 2.815 with p = 0.005, hence we reject the null hypothesis and conclude that in the universe of populations comparable to those in the analysis, the mean effect size is not precisely zero. The Q-value is 17.807 with 6 degrees of freedom and p = 0.007, thus we reject the null hypothesis that the true effect size is the same in all these studies. The I-squared statistic is 66%, which suggests that some 66% of the variance in observed effects reflects variance in true effects rather than sampling error. Assuming that the true effects are normally distributed, we can estimate that the prediction interval is -0.930 to 2.548. The small number of studies may limit the reliability of the analysis.

### Adaptive SIRT1 response to exercise

**Figure 4:**
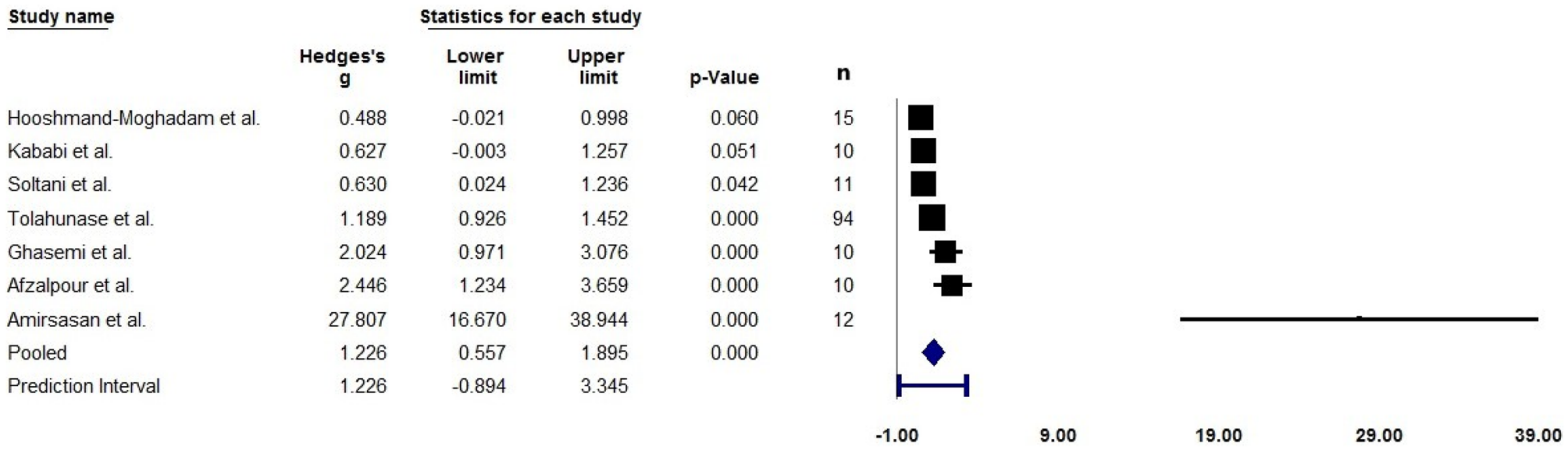
Forest plot quantifying SIRT1 levels in blood (measured via ELISA) after exercise training. Adjusted standardised mean difference (Hedges’ g), relative weight of each acute study response, confidence interval (diamond), and prediction interval (blue line) are also shown. n = sample size.

**Figure 5:**
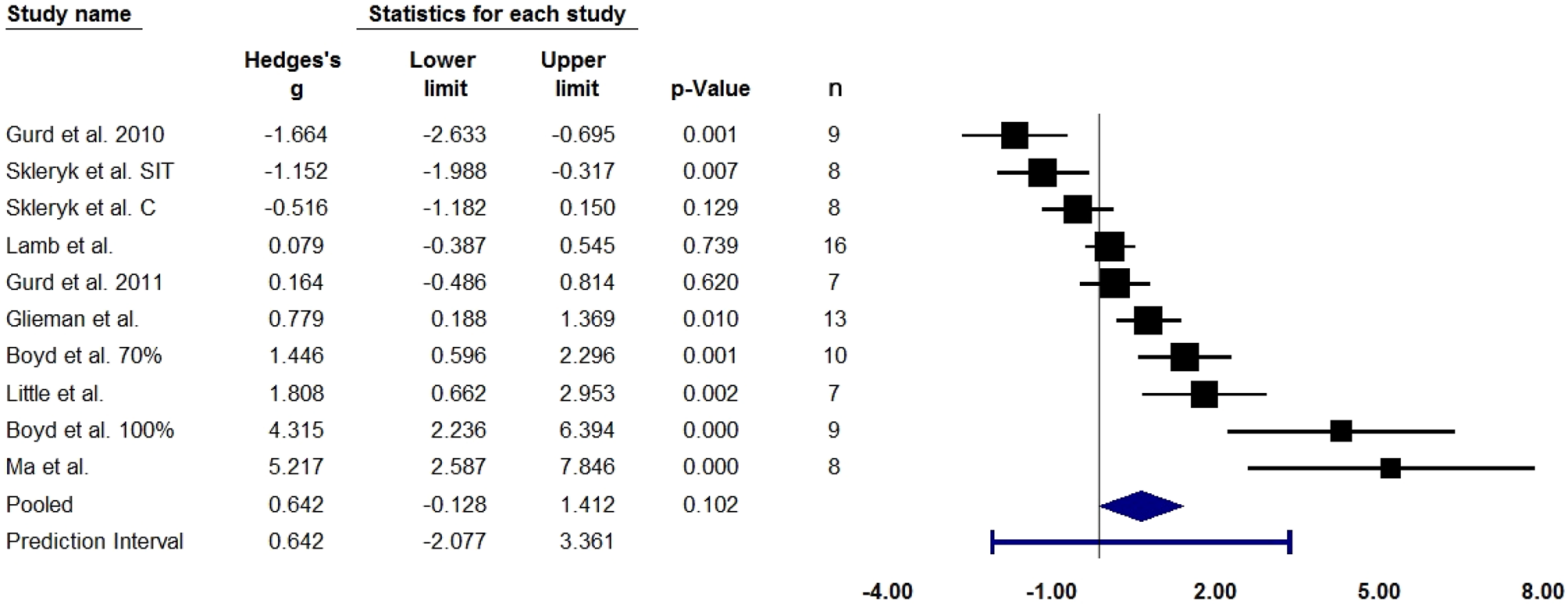
Forest plot quantifying SIRT1 protein content (Western blot) in skeletal muscle after exercise training. Adjusted standardised mean difference (Hedges’ g), relative weight of each acute response study, confidence interval (diamond), and prediction interval (blue line) are also shown. *C* cycling, *SIT* sprint interval training. Percentages denote exercise intensity. n = sample size.

A limited number of studies showed an increase in SIRT1 levels in blood (measured via ELISA) and increased SIRT1 activity in muscle (measured via fluorometric or sirtuin activity assay) after exercise training. The small number of studies may limit the reliability of the meta-analysis. Meanwhile, SIRT1 protein content in skeletal muscle (measured via Western blot) did not reach statistical significance. Figures 4 and 5 summarise the studies.

#### Blood SIRT1 levels after training

The analysis is based on seven studies (n = 162 participants) and utilised a random-effects model. The mean effect size adjusted with Hedges’ g is 1.226 with a 95% confidence interval of 0.557 to 1.895. The Z-value is 3.592 with p < 0.001, while the Q-value is 39.873 with 6 degrees of freedom and p < 0.001. The I-squared statistic is 85%, which suggests that some 85% of the variance in observed effects reflects variance in true effects rather than sampling error. Assuming that the true effects are normally distributed, we can estimate that the prediction interval is − 0.894 to 3.345.

With analysing resistance training exercise studies alone (4 studies, n = 131 participants), the mean effect size adjusted with Hedges’ g is 0.987 with a 95% confidence interval of 0.001 to 1.973. The Z-value is 1.962 with p = 0.05, while the Q-value is 29.474 with 3 degrees of freedom and p < 0.001. The I-squared statistic is 90%, and the prediction interval is − 3.221 to 5.195.

#### Muscle SIRT1 activity after training

There are only three studies (N = 32 participants) quantifying SIRT1 activity in muscle (via fluorometric or a sirtuin activity assay) after exercise training. The mean effect size adjusted with Hedges’ g is 1.476 with a 95% confidence interval of 0.464–2.487. The Z-value is 2.860 with p = 0.004, while the Q-value is 7.262 with 2 degrees of freedom and p = 0.026. The I-squared statistic is 72%, and the prediction interval is -9.990 to 12.941. The small number of studies may exaggerate the range of prediction interval.

#### Muscle SIRT1 content after training

The analysis is based on ten studies and utilised a random-effects model. The mean effect size adjusted with Hedges’ g is 0.642 with a 95% confidence interval of − 0.128 to 1.412. The Z-value is 1.634 with p = 0.102, hence we accept the null hypothesis. Nine out of 10 studies used high-intensity aerobic training, while one used resistance training. Analysing high-intensity aerobic training studies alone (9 studies) does not reach statistical threshold.

## Discussion

The main purpose of this exploratory review was to summarise and systematically assess published exercise interventions measuring SIRT1 (protein content, gene expression, enzyme levels, enzyme activity) in apparently healthy participants. Specifically, we wanted to know if and what type of exercise can increase SIRT1. A single bout of high-intensity or fasted exercise was shown to increase skeletal muscle *SIRT1* gene expression as measured by qPCR or RT-PCR, while repeated exercise training enhances blood SIRT1 levels as measured by ELISA. A limited number of studies (3, N = 32 participants) also observed an increase in muscle SIRT1 activity following exercise training. Overall, we determine that exercise acutely upregulates muscle *SIRT1* gene expression and chronically increases blood enzyme concentration. To our knowledge, this is the first systematic review and meta-analysis on SIRT1 response to exercise. Based on data from 34 studies (13 acute response, 21 training interventions), we highlight several novel outcomes.

Firstly, high-intensity or fasted exercise immediately upregulates *SIRT1* gene expression in human skeletal muscle. The said studies measured *SIRT1* expression using qPCR or RT-PCR from 0 to 3 h after a single bout of exercise. Immediate SIRT1 upregulation with PGC-1α deacetylation through protein kinase A (PKA) has been shown in C2C12 myotubes following adrenergic administration^57^. Although not in an exercise context, this demonstrates that hormonal signals may directly and rapidly modulate SIRT1 deacetylase activity, which can subsequently be sustained through an elevation in AMPK-dependent NAD^+^^58^. Cardiac muscle contraction and catecholamines, which are released during intense exercise, can activate PKA, perhaps explaining the immediate SIRT1 activation in high-intensity exercise^59^. In addition, PKA is activated when plasma glucose concentration falls below 65–70 mg/dL, which may explain the immediate increase in SIRT1 while in the fasted exercise state^60^.

SIRT1 levels increase with exercise intensity^26^ but only up to a certain point. One study found that supramaximal exercise (133% peak work rate) resulted in less expression of the SIRT1 target *PGC-1α* but a larger expression in early growth response 1 (*EGR1*), which is also known to induce *SIRT1* as a response to mechanical stretch in skeletal muscle^28^. This stretch-induced activation of *SIRT1* leads to deacetylation of FOXO4 and upregulation of superoxide dismutase which scavenges excess ROS^61^. EGR1 and SIRT1 form a negative-feedback loop, which may explain the decreased *SIRT1* expression at supramaximal exercise^61^. Supramaximal exercise also increases DNA damage, which recruits PARPs that use NAD and compete with SIRT1^22^.

High-intensity exercise increases muscle fibre recruitment and ATP turnover, which activates AMPK and SIRT1 activity; the latter by increasing cellular NAD^+^^61^. In support, a study in mice observed AMPK-dependent increases in NAD^+^ and PGC-1α deacetylation 3 h after resistance running, however, SIRT1 was not directly measured^62^. In young and fit individuals, but not in old and unfit, a single bout of cycling (20 min at 70% VO_2max_) can release extracellular NAMPT (eNAMPT) into the circulation, which increases NAD and SIRT1 within 1 h when administered to skeletal muscle cells^63^. Interestingly, injecting eNAMPT into muscle cells from young mice can prolong health and lifespan in old mice^64^.

Two studies have examined blood SIRT1 levels (as measured via ELISA) after a single bout of exercise (moderate intensity and high-intensity) and both observed an increase following exercise^26,29^. An additional investigation demonstrated that blood SIRT1 activity (fluorometric assay) is also enhanced following a single bout of exhaustive cycling^35^. With regards to repetitive exercise training, resting blood SIRT1 levels (ELISA) has also been shown to increase; albeit based on a moderate number of studies (7, n = 162 participants). The aforementioned studies involved resistance training and high-intensity aerobic training, which can both improve body composition, a factor that has been associated with increased circulating SIRT1 in larger long-term studies^18,65^. Circulating SIRT1 is negatively associated with fat mass, leptin, and insulin resistance, and positively associated with adiponectin, an “anti-obesity” hormone^66,67^. Interestingly, leptin and adiponectin are regulated partially by SIRT1 through PPAR-α and the circadian cycle^8,68^.

Low circulating SIRT1 is also associated with ageing-related disorders and has been proposed as a biomarker for Alzheimer’s and Parkinson’s disease^69,70^. In senior participants, two studies measured blood SIRT1 levels after resistance training, while another measured blood SIRT1 activity after resistance training; all demonstrating an increase post training^47,55,56^. Acutely, high-intensity aerobic exercise can also increase skeletal muscle *SIRT1* expression in seniors^36^.

SIRT1 levels in skeletal muscle, however, may not always increase after exercise training. It is also possible that SIRT1 muscle protein content does not increase until after several months of training, and several of the observed studies implemented less than 1 month of training. Several post-translational protein modifications may also regulate skeletal muscle SIRT1 adaptation to exercise. One of the studies found increased NAD, NAMPT, global sirtuin activity, and mitochondrial density, but no increase in skeletal muscle SIRT1 protein content after 10 weeks of resistance training, suggesting that it may be NAD or NAMPT levels, rather than SIRT1 protein content, that contributes to increased SIRT1 activity^49^.

There are only three published studies that quantified skeletal muscle SIRT1 activity through a fluorometric or sirtuin activity assay-based approach after exercise training. Although the meta-analysis reached statistical significance, it is limited by the small sample (n = 32). Only one study measured SIRT1 activity in blood, with significant effects after resistance training observed^56^. Muscle SIRT1 activity, rather than content, has been associated with mitochondrial biogenesis during exercise^46^. Meanwhile, blood SIRT1 activity has been positively associated with basal metabolic rate^71^. Thus, SIRT1 activity may be more responsive to exercise training. It is therefore suggested that any future studies in this exciting domain of investigation should consider measuring SIRT1 activity as well as expression.

Most studies documented in our analysis utilized high-intensity aerobic exercise protocols and young participants, with several using resistance training in aged and overweight/obese participants. A genuine attempt was made for this review to be exhaustive and group all eligible studies according to biomarker, exercise protocol, and participant characteristics. However, there were limited studies on old and overweight/obese groups to reach statistical significance. Hence, all age and weight groups (young, old, overweight, obese, normal) as well as training levels (trained, untrained, athletes, sedentary) were combined. We are aware that ageing and overweight participants may have heightened inflammatory profiles that may affect the exercise response. Moreover, athletes may have a blunted response to exercise due to their enhanced fitness. It is also conceivable that differences due to sex in the exercise-induced SIRT1 response may also exist, particularly aligned to muscle fiber type composition, given that SIRT1 expression is higher in female Type I muscle fibers.

One major limitation of this study is the unclear risk of bias in exercise related studies due to failure of reporting randomisation, concealment, and blinding protocols^72^. We used the original 11-item Cochrane Back Group Risk of Bias Tool that was partially utilized in a meta-analysis on exercise and DNA damage^22^. A subsequent validity analysis of the original tool determined that a summary score of 6 is the threshold for the separation of high-quality from low-quality studies^23^. Hence, and in line with a previous study, lower quality is classified with a score of 0–5, while the 6–11 range confirms higher quality studies^24^. Studies scoring 6 or higher are thus within the quality threshold. It is however, important to note, that a number of items in the current Cochrane tools repertoire are nearly impossible to implement in exercise focused studies, such as blinding and randomisation (e.g., in a repeated measures design, acute response studies where pre and post-exercise cannot be interchanged). As such, this can be interpretated that in fact all exercise studies are inherently at risk for performance bias. Comprehensive and recent work by Bonafiglia et al. suggests that given the apparent and perceived methodological issues when conducting a meta-analysis, future studies are encouraged to implement bias-reducing methodologies, such as the one used in the current work, and indeed the authors report on the various approaches that should be considered when attempting to mitigate performance bias in exercise studies^72^.

Another limitation of this study is that most data were extracted from figures rather than raw values using a graph digitizer software. However, to minimise error, highly accurate software was used based on other published work^73^.

We conclude that high-intensity exercise has a relatively small effect in acutely increasing *SIRT1* gene expression in skeletal muscle, while the effect observed in fasted exercise appears to be larger. Exercise training in general, or resistance training alone, has a large effect on resting blood SIRT1 levels, while a limited number of studies provide an evidence base for an increase in resting SIRT1 activity in skeletal muscle following exercise training. Taken together, these results reiterate the potential of exercise in prolonging health, partly due to upregulating SIRT1 acutely and chronically. Training variables such as intensity, adding resistance, or feeding status can be adjusted to maximise the health benefits of exercise. More studies examining SIRT1 activity following exercise and incorporating aged and overweight/obese populations are warranted.

## Methods

### Search strategy

Following PRISMA (Preferred Reporting Items for Systematic Reviews and Meta-Analyses) guidelines, a comprehensive article search was conducted using the keywords “exercise AND SIRT1” from March 1–31, 2022, using five databases: Scopus, PubMed, Embase, MEDLINE, and Web of Science. Search results were filtered to include only human trials, in peer-reviewed journals, and in English language. New studies published after the said dates were also added. Our search protocol was registered and published on PROSPERO (CRD42023427141).

### Inclusion/exclusion criteria

Articles were checked for the following inclusion criteria: (1) full report published in a peer-reviewed journal, (2) involving healthy adults, (3) controlled trial, and (4) with measures of SIRT1 (gene expression, protein content, enzyme levels, enzyme activity) in blood or skeletal muscle before and after exercise in acute response studies, or before and after training in intervention studies.

Studies with independent variables other than exercise (e.g., supplementation, diet, etc.) were included providing they consisted of a control group that received exercise alone, and only data from exercise-only groups were included in the meta-analysis.

“Training intervention studies” were those that implemented repeated exercise for a minimum of 2 weeks, which is the shortest intervention among the included studies. Meanwhile, “acute response studies” were those that conducted a single bout of exercise. “Fed state” suggests normal diet with the recommended macronutrient ratio (45–65% carbohydrates, 20–35% fats, 10–35% protein), while “fasted state” suggests 8–12 h overnight fasting which is a normal practice in exercise related studies. As such, data from participants doing more than 12 h fasting and special diets (e.g., vegan diet, high-carbohydrate diet, etc.) were excluded. Retracted articles were also excluded. The inclusion/exclusion criteria are shown in Table 4.

**Table 4:** Inclusion/exclusion criteria.

Criteria	Include	Exclude
Participants	Adults with no known disease	Children, adults with disease, animals
Exercise protocol	Aerobic and resistance exercise	Stretching, breathing, etc
Nutritional status (for acute response studies)	Fed state (normal diet and macronutrient ratio) or fasted state (8–12 h overnight fast)	High-fat, high-carbohydrate, high-protein, vegan diet, fasting for more than 12 h
Sample	Blood or skeletal muscle tissue	Neurons, adipocytes, etc
Outcome measure	SIRT1 gene expression, protein content, or enzyme levels	All other measures of SIRT1

### Data extraction

Articles that met the inclusion criteria were printed and summarised in a table following PICO (Patient, Intervention, Comparison, Outcome) guidelines and were grouped according to tissue sample (muscle vs blood), biomarkers of SIRT1 (gene expression, protein content, enzyme levels, enzyme activity), participants’ nutritional status (fed vs fasted), exercise type and intensity (see Table 1). In case of missing data (e.g., nutritional status of participants, average intensity of exercise protocol), authors were contacted via email to clarify information. Means, standard deviations (SD), and standard errors (SE) were extracted from full texts and figures using GetData Graph Digitizer software^73^.

### Quality assessment

Primary outcomes were defined as pre- and post-exercise SIRT1 measures (gene expression, protein content, enzyme levels, or enzyme activity). Risk of bias was assessed using the original 11-item Cochrane Back Group Risk of Bias Tool, namely: randomisation, concealment, baseline differences, patient blinding, care provider blinding, outcome blinding, co-intervention, compliance, dropouts, timing, and intention to treat (i.e., whether all participants were included in the analysis regardless of compliance). This approach was based on a related meta-analysis on exercise-induced DNA damage in healthy participants^22^. Studies were scored from 1 to 11, with a score of 6 distinguishing low from high quality studies; aligned to a validity study by Van Tulder et al.^23^. In practice, Paige et al. considered a score of 0–5 as “lower quality”, while a score of 6–11 was considered “higher quality”^24^. Table 5 summarises the quality assessment for risk of bias.

**Table 5:** Quality assessment for risk of bias using the Cochrane back group risk of bias tool.

Study	Randomisation	Concealment	Baseline similar	Patient blinding	Care provider blinding	Outcome blinding	Co-intervention avoided	Compliance acceptable	Dropout acceptable	Timing similar	Intent to treat	Total score
Afzalpour et al., 2017^38^	Yes	No	Yes	No	No	No	Yes	Yes	Yes	Yes	Yes	7
Aird et al., 2021^25^	Yes	No	Yes	No	No	No	Yes	Yes	Yes	Yes	Yes	7
Alfieri et al., 2015^39^	Yes	No	Yes	No	No	No	Yes	Yes	Yes	Yes	Yes	7
Amirsasan et al., 2019^40^	Yes	Yes	Yes	Yes	Yes	Yes	Yes	Yes	Yes	Yes	Yes	11
Boyd et al., 2013^41^	No	No	Yes	No	No	No	Yes	Yes	Yes	Yes	Yes	6
Cho et al., 2022^26^	Yes	No	Yes	No	No	No	Yes	Yes	Yes	Yes	Yes	7
Dimauro et al., 2016^42^	Yes	No	Yes	No	No	No	Yes	Yes	Yes	Yes	Yes	7
Dumke et al., 2009^27^	Yes	No	Yes	No	No	No	Yes	Yes	Yes	Yes	Yes	7
Edgett et al., 2013^28^	Yes	No	Yes	No	No	No	Yes	Yes	Yes	Yes	Yes	7
Ghasemi et al., 2020^29^	Yes	No	Yes	No	No	No	Yes	Yes	Yes	Yes	Yes	7
Gliemann et al., 2013^43^	Yes	Yes	No	Yes	Yes	Yes	Yes	Yes	Yes	Yes	Yes	10
Granata et al., 2020^30^	No	No	Yes	No	No	No	Yes	Yes	Yes	Yes	Yes	6
Gray et al., 2018^44^	No	No	Yes	No	No	No	Yes	Yes	Yes	Yes	Yes	6
Guerra et al., 2010^31^	Yes	No	Yes	No	No	No	Yes	Yes	Yes	Yes	Yes	7
Gurd et al., 2010^45^	No	No	Yes	No	No	No	Yes	Yes	Yes	Yes	Yes	6
Gurd et al., 2011^46^	No	No	Yes	No	No	No	Yes	Yes	Yes	Yes	Yes	6
Hooshmand al., 2020^47^	Yes	No	Yes	No	No	No	Yes	Yes	Yes	Yes	Yes	7
Kababi et al., 2022^48^	Yes	No	Yes	No	No	No	Yes	Yes	Yes	Yes	Yes	7
Lamb et al., 2020^49^	No	No	Yes	No	No	No	Yes	Yes	Yes	Yes	Yes	6
Little et al., 2010^50^	No	No	Yes	No	No	No	Yes	Yes	Yes	Yes	Yes	6
Ma et al., 2013^51^	No	No	Yes	No	No	No	Yes	Yes	Yes	Yes	Yes	6
Margolis et al., 2017^32^	Yes	Yes	Yes	Yes	Yes	Yes	Yes	Yes	Yes	Yes	Yes	11
Morales et al., 2013^33^	Yes	Yes	Yes	Yes	Yes	Yes	Yes	Yes	Yes	Yes	Yes	11
Mendham et al., 2016^34^	Yes	No	Yes	No	No	No	Yes	Yes	Yes	Yes	Yes	7
Potthast et al., 2020^35^	No	No	Yes	No	No	No	Yes	Yes	Yes	Yes	Yes	6
Radak et al., 2011^36^	No	No	Yes	No	No	No	Yes	Yes	Yes	Yes	Yes	6
Scribbans et al., 2014^52^	Yes	Yes	Yes	Yes	Yes	Yes	Yes	Yes	Yes	Yes	Yes	11
Skelly et al., 2017^37^	No	No	Yes	No	No	No	Yes	Yes	Yes	Yes	Yes	6
Skleryk et al., 2013^54^	Yes	No	Yes	No	No	No	Yes	Yes	Yes	Yes	Yes	7
Soltani et al., 2018^53^	Yes	No	Yes	No	No	No	Yes	Yes	Yes	Yes	Yes	7
Tolahunase et al., 2017^55^	No	No	Yes	No	No	No	Yes	Yes	Yes	Yes	Yes	6
Wasserfuh et al., 2021^56^	Yes	No	Yes	No	No	No	Yes	Yes	Yes	Yes	Yes	7

### Statistical analysis

#### Assessment of effect size

The Comprehensive Meta-Analysis (Version 4, NJ: USA: Biostat, Inc.) software was used to calculate random effects from means, standard deviations, and standard errors extracted from each article. Standardized mean difference (SMD) adjusted with Hedges’ *g* at 95% CI was calculated as the difference in means before and after exercise divided by the pooled standard deviation. SMD was used to express effect size, which was assessed using Cohen’s categories: 0.2–0.5 = small, 0.5–0.8 = medium, and > 0.8 = large. The overall effect size was assessed using Z-values with a significance level of *p* < 0.05.

#### Assessment of heterogeneity

The Q-statistic was used to test the null hypothesis that the true effect size is the same in all these studies, where *p* value ≤ 0.10 was considered significant heterogeneity. The I-squared (*I*^*2*^) statistic was used to express the proportion of the variance in observed effects that reflects variance in true effects rather than sampling error^74^.

#### Publication bias

Publication bias was assessed by visually analysing funnel plots, with a caveat that funnel plots may not be appropriate for small studies^75^. Both observed values and imputed values were plotted whereby imputed studies are perceived to be primarily negative in origin, while actual studies are regarded as being more positive (see Supplementary Materials S1).

### Supplementary Information

Supplementary Information.

## Data Availability

All data presented in this review were taken from the cited studies which are publicly available. Standardised values are shown in the forest plots, while funnel plots are provided in the Supplementary Materials.
