# Supplementary material for: A systematic review and meta-analysis of the SIRT1 response to exercise
Source: Sci Rep. 2023 Sep 7;13:14752. doi: 10.1038/s41598-023-38843-x (PMC10485048; doi:10.1038/s41598-023-38843-x)
Supplement: Supplementary file 1 — Supplementary Information. [file 41598_2023_38843_MOESM1_ESM.pdf]

A systematic review and meta-analysis of the SIRT1 response to exercise

Ciara Gallardo Juan[1]*, Kyle B. Matchett[2], Gareth W. Davison[1]

Supplementary Materials

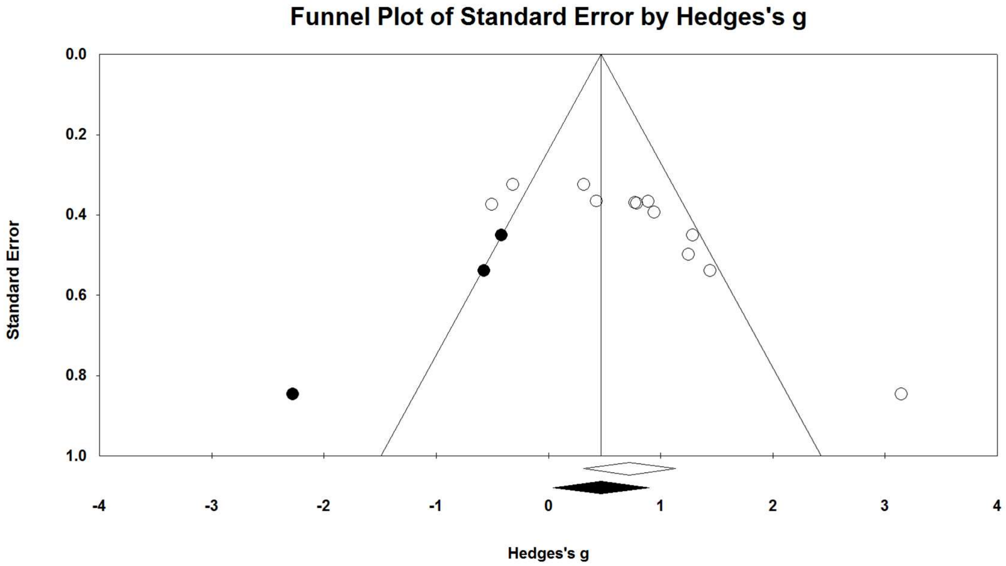

Studies measuring skeletal muscle *SIRT1* gene expression after high-intensity exercise (see Figure 2). Actual studies in white, imputed studies in black.

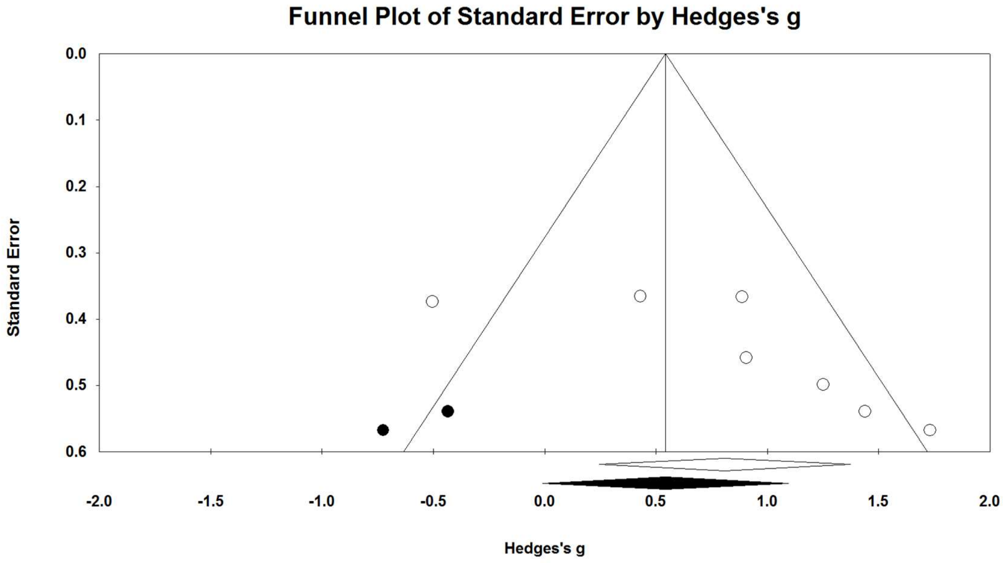

Studies measuring skeletal muscle *SIRT1* gene expression after fasted exercise (see Figure 3). Actual studies in white, imputed studies in black.

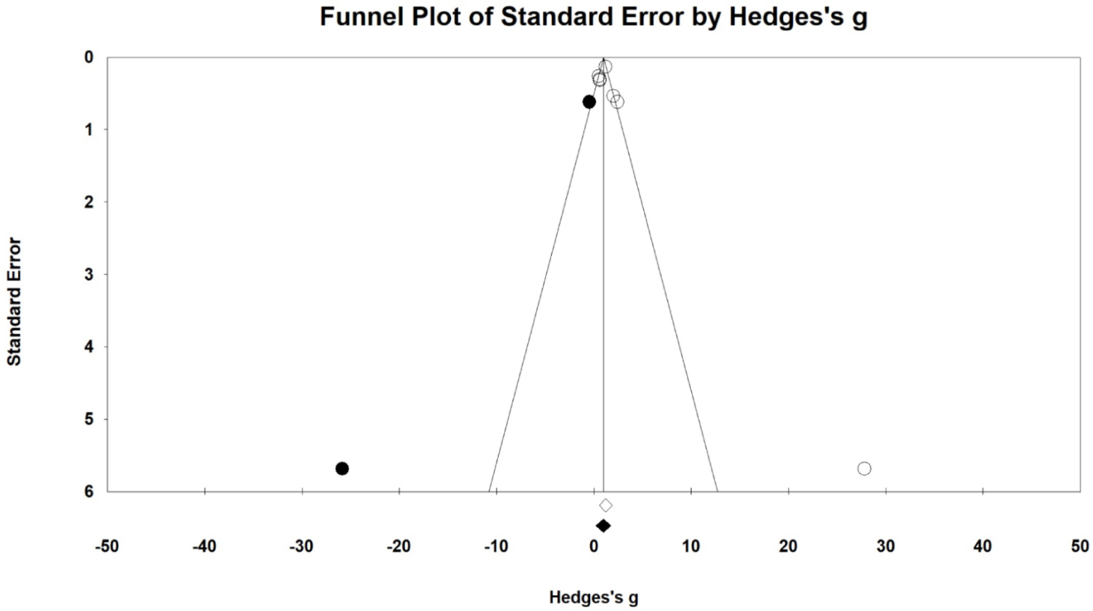

Studies measuring SIRT1 levels in blood (see Figure 4). Actual studies in white, imputed studies in black.

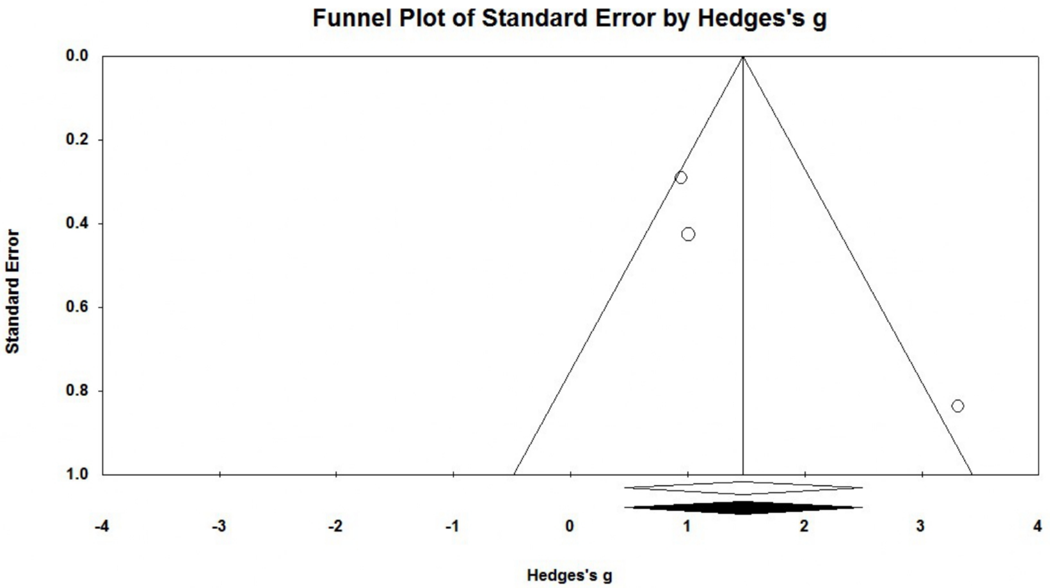

Studies measuring SIRT1 activity in skeletal muscle. Actual studies in white, imputed studies in black.

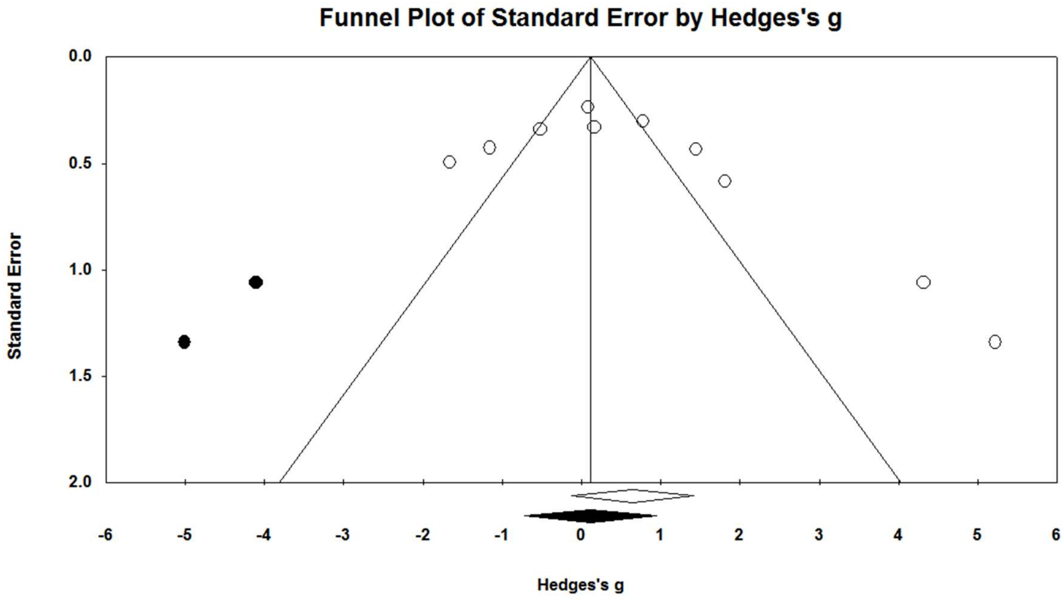

Studies measuring SIRT1 content in skeletal muscle (see Figure 5). Actual studies in white, imputed studies in black.
